# New microsatellite markers distinguish two species of ramps (*Allium tricoccum* Aiton Complex, Amaryllidaceae) and show variation in clonality and genetic diversity between species and among populations

**DOI:** 10.1371/journal.pone.0332086

**Published:** 2025-10-08

**Authors:** Sarah E. Nilson, Matt C. Estep, Eric P. Burkhart, Harvey Ballard, Ezra Houston, Bina S. Sitepu, Haley Velemirovich, Malia Costa

**Affiliations:** 1 Department of Biology, The Pennsylvania State University, Monaca, Pennsylvania, United States of America,; 2 Department of Biology, Appalachian State University, Boone, North Carolina, United States of America,; 3 Department of Ecosystem Science and Management, The Pennsylvania State University, University Park, Pennsylvania, United States of America,; 4 Department of Environmental and Plant Biology, Ohio University, Athens, Ohio, United States of America,; 5 Research Center for Ecology and Ethnobiology, National Research and Innovation Agency, Cibinong, West Java, Indonesia; Nuclear Science and Technology Research Institute, IRAN, ISLAMIC REPUBLIC OF

## Abstract

A ramp (*Allium tricoccum* Aiton), or wild leek, is a perennial herbaceous plant native to the forests of eastern North America. Like other members of the *Allium* genus, ramps produce sulfur-containing compounds that give them culinary and medicinal appeal. Ramps reproduce clonally via bulb division and sexually via seed production, but little is known regarding how much genetic diversity is present in ramps and ramp populations. Furthermore, there is an unresolved question regarding species delineation, with as many as four species suggested. We developed four polymorphic microsatellite markers that we used to measure genetic diversity in ramps and found that ramp populations have low to moderate levels of genetic variation and high differentiation and that individual ramp populations vary in clonality and genetic diversity. Finally, we provide the first preliminary genetic evidence supporting the delineation of the purported second ramp taxon, narrow-leaf ramps (*Allium burdickii* (Hanes) A.G. Jones)*.*

## Introduction

Ramps, also known as wild leeks (*Allium tricoccum* Aiton), are perennial herbs occurring most commonly on mesic forestlands in the midwestern and eastern regions of North America [1–3]. Ramps belong to the genus *Allium* L., which includes onion (*A. cepa* L.), garlic (*A. sativum* L.), leek (*A. ampeloprasum* L.), and other economically important plants. Containing 550–800 species [4–5], *Allium* was traditionally submerged in the broadly circumscribed Liliaceae, *sensu lato*, but molecular phylogenetic studies and treatments now place it in Alliaceae [6] or Amaryllidaceae [7–8].

Increased public interest in ramp collection (i.e., foraging) from wild populations for personal and commercial consumption, along with an increase in commercial trade, has raised conservation concerns over the impact of wild collection in some parts of the range because ramp populations can be slow to recover following harvest [9–10]. Due to these concerns, ramp collection has been restricted to personal use only in some regions of the United States [e.g., 11] and Canada [e.g., 12]. As ramps have become increasingly commercialized, they are now being promoted as a specialty crop for use within agroforestry systems, especially through the practice known as forest farming [2,13]. This cropping system has potential to be both a conservation mechanism and an economic development opportunity [2,14,15].

Ramps emerge from bulbs in the late winter and early spring, before forest canopy leaf-out, to take advantage of higher insolation in the forest understory [16–17]. During the approximately six-week photosynthetic phase, ramp bulbs swell [17–18] and may also begin clonal division [19]. During the summer months, the scape bears small, white flowers arranged in a narrowly conical to nearly spherical umbel. Ramps can reproduce via both self-compatibility and out-crossing [20–21]. Following pollination, the seeds mature in three-seeded capsules during late summer and early fall before the plant enters winter dormancy. One demographic study of *A. tricoccum* found that sexual reproduction contributes minimally to population growth and that asexual reproduction, a process which can take five to eight years, is the primary mode of population maintenance [19]. However, significant annual variation in seed production and seedling recruitment was also noted [19].

Despite the growing interest in ramps as a wild food and agroforestry crop [13], information regarding ramp genetic diversity remains limited. Genetic diversity gives populations and species the potential to adapt to and persist in changing conditions, such as those brought on by disturbance or climate change [22–24]. Although counter-intuitive, clonal and partially clonal plant populations do have the potential to maintain moderate to high levels of genetic diversity, depending on the amount of diversity present in the founding population, frequency of seedling establishment, and the degree of gene flow into the population via pollen or seed dispersal [25]. The only study of ramp genetic diversity to date used seven isoenzyme systems to document genetic variation in ramps collected at six sites in Quebec [26]. Only two of the seven isoenzyme markers were polymorphic, which led the researchers to conclude that ramp genetic diversity is very low, and that ramps are likely reproducing primarily via clonal reproduction [26]. Since the original study was performed, ramp harvesting has increased and become commercialized [13], creating a need to develop better genetic tools which can be used to measure genetic variation within and among ramp populations across the range of this species. These data can be used to identify populations of conservation concern, shed insight into the relative contributions of asexual and sexual reproduction in ramps at the population and species levels, and help land managers and forest farmers develop ramp harvesting and stewardship guidelines to support ramp conservation.

The development of tools to assess ramp genetics may also help resolve the question of how many “ramp” taxa exist in North America. There has been debate as to whether there is one polymorphic species (*A. tricoccum*), one species with two varieties (var. *tricoccum* and var. *burdickii* Hanes) or two distinct species (*A. tricoccum* and *A. burdickii* (Hanes) A.G. Jones) in eastern North America. The existence of two ramp taxa was first proposed by Hanes and Ownbey [27], when they contrasted the *burdickii* race against *A. tricoccum* based on morphological, phenological, and habitat differences in specimens from Michigan and Wisconsin. Hanes [28] later formally described var. *burdickii*. Walker [29] examined populations in New York state and confirmed observations of two taxa. Jones [30] conducted extensive taxonomic studies of the *Allium tricoccum* complex and documented many macromorphological trait differences and mostly non-overlapping phenologies between the two taxa. Jones concluded that the two taxa represented morphologically, ecologically, and phenologically different species and raised var. *burdickii* to species rank, as *A. burdickii* (Hanes) A. G. Jones.

Kauffman [31] and McNeal and Jacobsen [32] retained *A. burdickii* as a variety of *A. tricoccum.* Kauffman noted that some specimens appeared to be intermediate or expressed confounding variation patterns that did not fit either taxon, while McNeal and Jacobsen stated that *A. burdickii* expressed overlapping traits and geographic range with *A. tricoccum* and was doubtfully distinct. Bell [20] conducted herbarium and field studies like those of Jones [30], but they initially separated plants into two groups based solely on presence or absence of red-purple pigmentation on any part of a plant. Bell also made laboratory observations of morphology and conducted crossing experiments among individuals, populations, and taxa to interpret inter-fertility on living plants from populations in eastern West Virginia, Pennsylvania, and New York. They reported significant overlap of morphological traits and geographic distributions, as well as complete interfertility, between the two taxa, and concluded that only one polymorphic species, *A. tricoccum*, should be recognized.

The latest taxonomic study of the *A. tricoccum* complex, by Sitepu [33], examined living plants retrieved from two dozen sites in eight midwestern and Appalachian states. Plants grown in a common garden were observed over two seasons for morphological traits and phenologies following the approach of Jones [30]. Sitepu [33] documented noticeable changes in pigmentation in individuals of *A. tricoccum sensu lato* over seasons in Athens County, Ohio populations, and confirmed the infrequent presence of entirely unpigmented individuals of *A. tricoccum* in some populations. Statistical analyses of morphological traits distinguished four taxa: *A. tricoccum sensu stricto*; *A. burdickii sensu stricto* from the easternmost Great Plains and Midwest; south green ramps in the Interior Low Plateau of Kentucky and Tennessee and possibly southern Ohio and Indiana; and highland green ramps in the Appalachian Mountains. Phenological studies [33] confirmed that the green ramps taxa initiated and concluded anthesis weeks earlier than *A. tricoccum* with rare overlap in blooming times. Sitepu [33] concluded that the *A. tricoccum* complex consisted of four distinct evolutionary species under the Unified Species Concept [34]. Aside from these varied and sometimes conflicting taxonomic studies, field botanists and naturalists have continued to report the occurrence of strictly unpigmented or weakly pigmented plants otherwise identical to typical *A. tricoccum*, populations that do not match any one taxon fully, and populations that express overlapping blooming times between *A. burdickii* sensu lato and *A. tricoccum*.

Recent floristic treatments support *A. burdickii sensu lato* as a separate species [3,33] (see S1 Fig for key differences). While *A. tricoccum* is found in northeastern United States (U.S.) and adjacent regions of eastern Canada and in the Great Lakes region, the distribution of *A. burdickii* in the broad sense has been suggested to occur from Maine to North Dakota and south of New Jersey [1,3,30,35]. However, further south, distribution maps for *A. burdickii* are not consistent on whether it is present or absent in Virginia and North Carolina [3,35,36], and before this research there were no documented occurrences of *A. burdickii* in Pennsylvania*. Allium tricoccum* has two different color extremes and can have either red/purple stems or green/white stems whereas *A. burdickii* only has green/white stems. The misidentification of *A. burdickii* as unpigmented (lacking anthocyanin) *A. tricoccum* and vice versa has likely contributed to unclear distributions of *A. burdickii.* The sole genetic study on ramps was not able to genetically distinguish *A. burdickii* from *A. tricoccum*, however the isoenzyme markers had limited resolution [26]. New genetic methods could allow us to determine if *A. burdickii* is a unique species. The two additional green/white taxa similar to *A. burdickii* proposed by Sitepu [33] raise further questions about the diversity of *A. burdickii* and its separation from *A. tricoccum*.

Microsatellite markers specific to *A. tricoccum* have not been developed previously because ramps are not as economically or agriculturally significant as some other *Allium* species. Microsatellites are short tandem repeats of DNA found throughout prokaryotic and eukaryotic genomes. Microsatellites have a high rate of evolution and are highly diverse in repeat length making them excellent markers for genetic studies. There are twenty-five existing microsatellite markers that have been developed among varieties of *A. fistulosum* (bunching onion), onion, and garlic that had potential for successful use in *A. tricoccum* [37–39]. All these markers were screened against a small number of individuals and found to not cross amplify well.

This study sought to 1) identify polymorphic markers that can successfully amplify *A. tricoccum* DNA, 2) utilize these markers to measure genetic diversity and clonality in ramps and ramp populations; and 3) examine ramp population structure, and determine if there is genetic support for more than a single ramp taxon

## Materials and methods

### Microsatellite marker development

High quality *A. tricoccum* DNA was sent to West Virginia University (Morgantown WV, USA) Core Facility for Illumina sequencing. The resulting sequences were trimmed in FastP for quality using default parameters [40]. MSATcommander was utilized to identify microsatellite loci and design primers to amplify these loci [41]. Primers were designed between 19 to 25 base-pairs long, GC content was between 45–55%, and a PIG-tail sequence [42] was added to reverse primers to reduce stutter. One hundred primer pairs were screened against a small number of individuals (7 DNA samples and a negative control) to assess consistent amplification, product size, and to identify polymorphisms. Eighteen primer pairs that showed consistent amplification and expected product size were further assessed by labeling the forward primer with a 5’ fluorochrome (either FAM, VIC, or NED) to facilitate multiplex PCR (S1 Table).

### Sampled sites, species identification, and voucher collection

**Fig 1:**
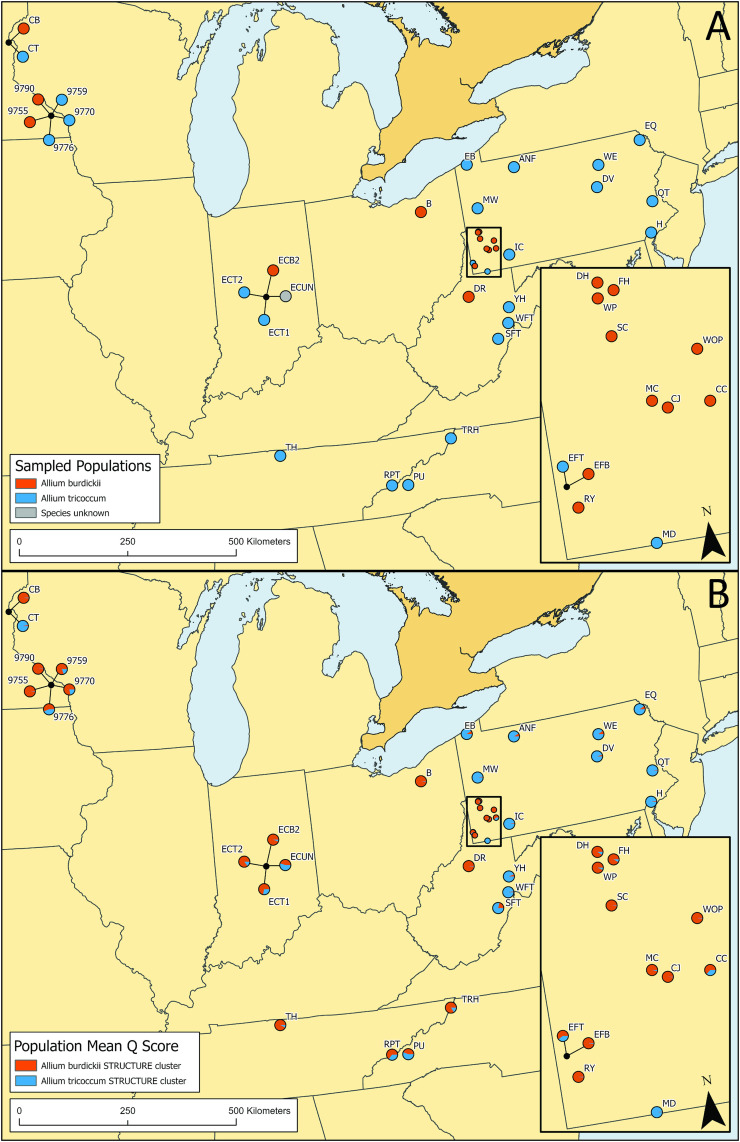
Locations of ramp collections and population mean Q scores from STRUCTURE. A, Sites were identified as either *A. burdickii* (orange) or *A. tricoccum* (blue) at collection. One site appeared to be a mixed collection *of A. burdickii* and *A. tricoccum* based on vouchers provided to the researchers and is shown in gray. Collections made at the same location are connected by black lines. B, STRUCTURE results from analysis of clone-corrected data set (n = 276 multilocus genotypes (MLGs) collected at 41 sites) based on four microsatellite markers. Population mean Q scores for K = 2 genetic clusters are denoted in pie graphs. MLGs collected at *A. burdickii* sites are found in the orange cluster (labeled *A. burdickii* STRUCTURE cluster), while the second cluster in blue, contained only MLGs collected at *A. tricoccum* sites (labeled *A. tricoccum* STRUCTURE cluster). Contains information licensed under the Open Government License – Canada.

Ramp sites were visited and sampled between 2019–2023 (Fig 1A and S2 Table). Where needed, several visits were made to sites to observe changes in phenology and morphology to make a positive confirmation of which species was being sampled (key differences between the taxa are shown in S1 Fig). In order to capture the breadth of genetic diversity at each site, approximately 20 ramps were randomly sampled per site with a minimum of 20 meters between samples. Typically, this was achieved by walking the perimeter of the site and sampling the edges of each population, and then making a transect through the center. In some cases, ramp populations were scattered across multiple hectares and an effort was then made to sample plants randomly throughout the site. For each sample, a single leaf was harvested and placed in a coin envelope and dried in silica gel. This study did not require IRB approval. The authors were given written and/or verbal consent by private landowners prior to sampling. Permits were not required for sampling on private land. Collection permits (USDA Forest Service 4080; PA DCNR State Forests Permit SFRA-1832, SFRA-1908, SFRA-2009; PA DCNR State Parks Permit 2019–69, 2023–30; PA DCNR Wild Plant Management Permits 17–035, 18–035, 19–035, 20–035, 21–035, 22–035, 23–035, 24–035, 20–761, 19–761, 18–761; and National Parks Service GRSM-2020-SCI-2047) were acquired for public lands prior to sample and voucher collection.

While most sites were sampled by the authors, some samples were provided by land managers and botanists who we determined were reliable sources with properly identified populations. If both species were believed to be present at the site, the species were collected separately. The species cooccurred at four localities in Minnesota (MN) (two sites), Indiana (IN), and Pennsylvania (PA). At two of these co-occurring sites the land managers who collected samples on our behalf made multiple collections from different areas of the site. We elected to keep these collections separate in the analysis. When permitted, voucher specimens were collected, pressed, mounted, and deposited at the Carnegie Museum of Natural History (CM), Ohio University (BHO), and Penn State University (PAC) herbaria. Based on voucher specimens, one site ECUN, appeared to be a mixed collection of *A. burdickii* and *A. tricoccum* and was not included in *A. burdickii* vs. *A. tricoccum* comparisons.

### Genomic DNA isolation

Dried leaf tissue (2–3 mg) was weighed and placed into a screw cap microtube with a sterilized steel bead. The tubes were placed into a Bead Bug (45 sec at 400x10) until the tissue was finely ground into a powder. Genomic DNA was isolated from the ground leaf tissue using the Thermo Scientific GeneJET Plant Genomic DNA Purification Mini Kit according to the manufacturer’s directions or using a modified CTAB method [43]. Extracted DNA was quantified with a Nanodrop-1000 Spectrophotometer and agarose gel electrophoresis. High-quality DNA samples were then diluted to 20 ng/ul and were randomly arrayed into 96-well plates.

### Amplification of microsatellite loci

Microsatellite loci were amplified using multiplex PCR using five different multiplex mixes containing primers to amplify three to four loci per mix (S1 Table). Each 15 µL reaction contained 7.5 µL of 2X Qiagen Type-it Microsatellite PCR Master Mix, 0.2uM of each primer and 2.5 ng/µL genomic DNA, and dd H2O. PCR was performed in Bio-Rad 96-well plates using MiniAmp™ Plus Thermal Cycler. The PCR cycle used was an initial denaturation of 95°C for 15 min, followed by 94°C denaturation for 30 sec, 58°C annealing for 90 sec, 72°C extension for 90 sec, for 35 cycles and a final extension at 72°C for 10 min, and hold at 4°C. Negative control wells containing no DNA were included in each PCR run. PCR products were diluted 1:25 using dd H_2_O. 1 µl of the diluted PCR product was added to 10 µL Hi-Di formamide (Applied Biosystems) containing 0.42 µL GeneScan 500 LIZ dye size standard (Applied Biosystems), denatured for 5 min at 37°C, and run on an Applied Biosystems 3730XL DNA Analyzer at the Penn State University Genomics Core Facility (State College, PA USA) to determine microsatellite allele sizes. Chromatographs were viewed and microsatellite allele sizes were scored using the microsatellite plug-in 1.4.7 for Geneious Prime 2023.1.2.

### Data analysis

Microsatellite and population genetic diversity statistics were calculated for each locus and site using GenAlEx 6.5 [44] and the *poppr* package for R [45]. Tests for Hardy-Weinberg equilibrium and the presence of null alleles were performed using *poppr*. Clones were identified and removed from populations using the clonecorrect command in *poppr* based on a genetic distance threshold informed by the observed distribution of pairwise genetic distances (S2 Fig), and diversity statistics were calculated for each site; when both species were present at a site, they were analyzed separately. Samples with missing genotype data were removed before clone counting. Spatial autocorrelation analysis was not feasible due to the lack of precise sub-meter spatial data. To assess the populations for linkage disequilibrium (LD), the index of association, rbard, and the p-value for the test of the null hypothesis of no LD between markers was performed in *poppr*, on both the non-clone-corrected and the clone-corrected data sets. Statistical comparisons between *a priori* determined *A. tricoccum* and *A. burdickii* sites were performed using non-parametric Mann-Whitney U test using GraphPad Prism 9.4.1.

Bayesian clustering analysis using STRUCTURE v.2.3.4 admixture model was used to examine population structure [46]. Two STRUCTURE models were run. The first was run on the entire data set (n = 593), including individuals with incomplete genotype data. The second analysis was performed on clone corrected data and any samples with missing genotype data were removed (n = 276). All STRUCTURE analyses were performed using the admixture model with assumed correlation between alleles. The number of most likelihood populations (K) was set from 1 to 9 with 10 iterations run per K value. 100,000 initial burn-in iterations were followed by 100,000 Markov Chain Monte Carlo (MCMC) replications. STRUCTURE results for multiple iterations of each K were aggregated and visualized using CLUMPAK [47]. The optimal number of clusters was determined using the Evanno Δ K method [48] using CLUMPAK [47]. Genotypes were assigned to the group that had the highest membership coefficient, as long as a coefficient (q) ≥70%, otherwise they were placed in a third group called admixed. Discriminant analysis of principle components (DAPC) was performed in R using clone corrected data on populations with 10 or more individuals with full genotype data. First a Principal Component Analysis was performed in *ade*4 v1.7-22 [49]. Then the number of clusters were identified and a DAPC was constructed using *Adegenet* v2.1.10 [50]. To test the robustness of the STRUCTURE and DAPC results, both analyses were repeated on the clone-corrected data using only three of the four loci (AT04 was dropped). To assess the distribution of genetic variation, Analysis of Molecular Variance (AMOVA) was performed using ARLEQUIN version 3.5.2.2 [51] with significance tests based on 1,000 permutations. Variation was partitioned hierarchically among *A. burdickii* and *A. tricoccum* groups, among populations within each group, and within populations. AMOVAs were performed on non-clone corrected data which included only individuals with complete genotype data and populations with ten or more individuals. The population ECUN, which appeared to be a mixed collection based on voucher specimens, was not included in the analysis. AMOVA on clone-corrected data could not be performed since some of the populations were entirely clonal and contained a single MLG.

## Results

### Microsatellite marker diversity

The eighteen primer pairs were initially tested for polymorphism on a subset of 95 individuals from 12 different sites across the sampled range and included both *A. tricoccum* and *A. burdickii samples*. While all primer pairs successfully amplified a product, only four loci (22.22%) were polymorphic (S1 Table). These four primer sets were used to genotype a total of 593 ramps collected at 41 sites, and complete genotype data was acquired for 576 individuals. The markers had moderate levels of variability and diversity and high evenness making them suitable for use in this study (Table 1). A total of 26 alleles were amplified and the total number of alleles per locus (Na) ranged from 3–9 alleles (mean = 6.5). Nei’s genetic diversity (He) ranged from 0.46 to 0.79 (mean = 0.64) and evenness (E5) ranged from 0.70 to 0.81 (mean = 0.77, Table 2). Simpson’s index (1949) was also calculated and was identical to He values for all markers. Tests for Hardy-Weinberg Equilibrium (HWE, S1 File) were performed separately for each marker at each site (152 tests total). Non-significant results were observed in 56 tests (36.8%) and significant deviation from HWE was found in 52 tests (34%). The allele was monomorphic in 44 tests (28.9%). Tests for null alleles based on homozygote excess indicated we cannot rule out the possibility of null alleles at each locus (S2 File).

**Table 1:** Microsatellite loci summary statistics.

Locus	Number of observed alleles	Nei’s gene diversity (H_exp_)	Evenness (E_5_)
AT04	3	0.46	0.81
AT32	5	0.59	0.70
AT55	9	0.79	0.79
AT58	9	0.74	0.77
Total	26		
Mean	6.5	0.64	0.77

**Table 2:** Population Summary Statistics.

Site	N	MLG	eMLG	SE	R	H	G	lambda	E.5	H_exp_	lambda (CC)	H_exp_ (CC)
***A. burdickii***												
9755	17	3	2.99	0.08	0.18	1.09	2.92	0.657	0.979	0.343	0.667	0.417
9790	15	5	4.00	0.75	0.33	1.23	2.71	0.631	0.707	0.252	0.800	0.250
B	14	1	1.00	0.00	0.07	0.00	1.00	0.000	NA	0.130	0.000	0.250
CC	17	3	2.43	0.59	0.18	0.58	1.44	0.304	0.559	0.260	0.667	0.533
CB	16	1	1.00	0.00	0.06	0.00	1.00	0.000	NA	0.129	0.000	0.250
ECB2	15	9	7.10	0.85	0.60	2.08	7.26	0.862	0.890	0.389	0.889	0.399
EFB	20	4	3.71	0.46	0.20	1.28	3.33	0.700	0.899	0.213	0.750	0.214
FH	15	2	1.67	0.47	0.13	0.25	1.14	0.124	0.512	0.162	0.500	0.333
MC	14	4	3.42	0.62	0.29	0.99	2.13	0.531	0.667	0.153	0.750	0.214
RY	15	7	5.57	0.84	0.47	1.71	4.59	0.782	0.794	0.276	0.857	0.305
SC	16	2	2.00	0.01	0.13	0.66	1.88	0.469	0.941	0.288	0.500	0.375
WP	16	2	1.62	0.48	0.13	0.23	1.13	0.117	0.504	0.129	0.500	0.125
WOP	10	2	2.00	0.00	0.20	0.61	1.72	0.420	0.860	0.291	0.500	0.375
**Mean**	**15.38**	**3.46**	**2.96**	**0.40**	**0.23**	**0.82**	**2.48**	**0.431**	**0.756**	**0.232**	**0.568**	**0.311**
***A. tricoccum***												
9759	14	2	1.93	0.25	0.14	0.41	1.32	0.245	0.64	0.064	0.500	0.167
9770	17	9	6.82	0.89	0.53	2.07	7.05	0.858	0.875	0.482	0.889	0.472
9776	16	6	4.74	0.80	0.38	1.49	3.56	0.719	0.745	0.468	0.833	0.477
ANF	14	14	10.0	0.00	1.00	2.64	14.0	0.929	1.000	0.493	0.929	0.493
CT	11	2	2.00	0.00	0.18	0.47	1.42	0.298	0.698	0.078	0.500	0.167
DV	14	12	8.85	0.69	0.86	2.40	9.80	0.898	0.874	0.303	0.917	0.322
ECT2	13	7	6.03	0.72	0.54	1.78	5.12	0.805	0.838	0.335	0.857	0.385
ECT1	13	8	6.61	0.78	0.62	1.89	5.45	0.817	0.797	0.311	0.875	0.365
EFT	16	3	2.50	0.57	0.19	0.60	1.47	0.32	0.571	0.157	0.667	0.267
EQ	14	10	7.65	0.83	0.71	2.14	7.00	0.857	0.796	0.358	0.900	0.368
EB	14	13	9.51	0.50	0.93	2.54	12.3	0.918	0.963	0.450	0.923	0.452
H	13	4	3.53	0.57	0.31	1.03	2.25	0.556	0.694	0.092	0.750	0.188
IC	13	12	9.42	0.49	0.92	2.46	11.3	0.911	0.961	0.401	0.917	0.418
MD	16	1	1.00	0.00	0.06	0.00	1.00	0.000	NA	0.000	0.000	0.000
MW	15	13	9.14	0.66	0.87	2.52	11.8	0.916	0.945	0.392	0.923	0.405
PU	10	9	9.00	0.00	0.90	2.16	8.33	0.880	0.952	0.565	0.889	0.567
QT	15	5	4.23	0.68	0.33	1.34	3.17	0.684	0.772	0.169	0.800	0.272
RPT	20	20	10.0	0.00	1.00	3.00	20.0	0.950	1.000	0.560	0.950	0.560
SFT	14	13	9.51	0.50	0.93	2.54	12.3	0.918	0.963	0.484	0.923	0.500
TRH	24	16	8.15	1.02	0.67	2.60	11.1	0.910	0.808	0.313	0.938	0.346
TH	14	6	5.34	0.64	0.43	1.67	4.90	0.796	0.901	0.231	0.833	0.330
WF	13	11	8.85	0.64	0.85	2.35	9.94	0.899	0.941	0.355	0.909	0.364
WE	14	14	10.0	0.00	1.00	2.64	14.0	0.929	1.000	0.439	0.929	0.439
YH	12	8	7.14	0.65	0.67	1.98	6.55	0.847	0.889	0.484	0.875	0.479
**Mean**	**14.54**	**9.08**	**7.16**	**0.50**	**0.63**	**1.86**	**7.71**	**0.744**	**0.853**	**0.333**	**0.809**	**0.367**
ECUN	13	9	7.53	0.75	0.69	2.10	7.35	0.864	0.888	0.555	0.889	0.542
**Total**	**562**	**215**	**9.35**	**0.78**	**0.38**	**4.72**	**56.40**	**0.982**	**0.498**	**0.645**	**0.994**	**0.624**

*N* is number of samples with complete genotypic data; MLG is number of observed multilocus genotypes; eMLG is expected number of MLG at the smallest sample size ≥10 based on rarefaction; SE is standard error based on eMLG; R is clonal richness defined as MLG-1/N-1, H is Shannon–Wiener index (1948) on MLG diversity; G is Stoddart & Taylor’s, 1988 index of MLG diversity; lambda is Simpson’s, 1949 index; E.5 is evenness; Hexp is Nei’s, 1978 unbiased gene diversity; lambda (CC) is lambda for clone-corrected data; Hexp (CC) is Hexp for clone-corrected data. Based on voucher specimens, site ECUN likely contained a mixture of *A. burdickii* and *A. tricoccum* individuals and is therefore reported separately.

### Population clonality and diversity

Two hundred and fifteen unique MLGs were detected (S3 Fig), 17% of MLGs were found at multiple sites while 83% were found at a single site (S4 Fig). Population summary statistics were calculated for sites with ten or more individuals with full genotype data (38 sites, n = 562). Clonality varied by site. Two *A. burdickii* sites (B, CB) and one *A. tricoccum* site were completely clonal consisting of a single MLG. Expected multi-locus genotypes, (eMLG, expected number of MLGs at the smallest sample size ≥10 based on rarefaction) ranged from 1–10 and clonal richness (R) ranged from 0.06 to 1.00 (Table 2). Compared to *A. tricoccum* sites, *A. burdickii* sites had significantly fewer eMLGs (*A. tricoccum* mean eMLG = 7.16 vs 2.96 for *A. burdickii*, p = 0.0003) and significantly lower Shannon Index (H, p = 0.0006). However, *A. tricoccum* sites varied in clonality, with eMLGs ranging from 1–10. In addition to a high frequency of identical genotypes, another expected consequence of clonal reproduction is non-random associations between loci [52–53]. Therefore, tests for LD are commonly employed to determine the extent of clonal reproduction in clonal species [53–54]. We were able to test for LD in populations with three or more MLGs (seven *A. burdickii* sites, and 21 *A. tricoccum* sites). The null hypothesis of no LD was rejected for three *A. burdickii* sites (9755, CC, ECB2) and two *A. tricoccum* sites (9776, YH) when using the non-clone-corrected data, but the tests were not significant when using the clone-corrected data (S3 Table) which suggests that these populations are reproducing both asexually and sexually.

Simpson’s index (lambda) and Nei’s unbiased gene diversity (He) were calculated for each collection and across all collections for both non-clone-corrected and clone-corrected data (Table 2). Across all sites (both *A. tricoccum* and *A. burdickii* collections) ramps had moderate levels of genetic diversity (lambda = 0.982, lambda CC = 0.994, Hexp = 0.645, HexpCC = 0.624). However, there was less diversity at the population level. *A. burdickii* sites had a significantly lower mean Simpson’s index (lambda = 0.431, lambda CC = 0.568) than *A. tricoccum* sites (lambda = 0.744, lambda CC = 0.809, p = 0.0007 for non-clone-corrected; p = 0.0005 for clone- corrected). Mean Nei’s genetic diversity was also lower for *A. burdickii* collections (Hexp = 0.232, HexpCC = 0.311) than for *A. tricoccum* collections (He = 0.333, HexpCC = 0.367) but the difference was only significant for the non-clone-corrected data (p = 0.02). In addition, *A. tricoccum* sites showed a greater range in genetic diversity (HexpCC ranged from 0.000 to 0.567) compared to *A. burdickii* sites (HexpCC ranged from 0.125 to 0.533). Genetic diversity was the lowest at *A. tricoccum* sites MD, 9759, CT, and H (HexpCC ranged from 0.000 to 0.188) and the highest at sites PU, RPT, SFT, ANF (HexpCC ranged from 0.493 to 0.567). Among *A. burdickii* sites, the lowest diversity was found at site WP (HexpCC = 0.125) and was the highest at site CC (Hexp = 0.533). The sites that had the most unique MLGs (not found at any other site) were RPT and WE (17 and 13 MLGs) for *A. tricoccum* and ECB2 and RY (9 and 5 MLGs) for *A. burdickii* (S4 Fig).

### Genetic structure

**Fig 2:**
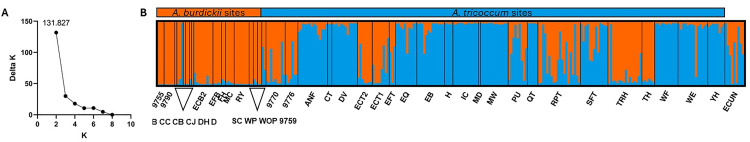
STRUCTURE results for clone-corrected data. A. Delta K values for K = 2-8 determined using the Evanno method [48]; B. CLUMPAK plot of STRUCTURE results for clone-corrected data set (n = 276 MLGs collected at 41 sites) based on four microsatellite markers for K = 2 organized by site. MLGs collected at *A. burdickii* sites correspond to one cluster (orange) with little admixture with the second genetic cluster (blue) except for one MLG collected at site CC which may have been misidentified at collection. The blue cluster contained only MLGs collected at *A. tricoccum* sites. Some MLGs collected at *A. tricoccum* sites were placed in the orange cluster or showed potential admixture with *A. burdickii*. Admixture was most prevalent at sites where *A. burdickii* occurred with *A. tricoccum* (9759, 9770, 9776, ECT2, ECT1, EFT, ECUN) and at southern sites (PU, RPT, SFT, TRH, TH).

Bayesian STRUCTURE, DAPC, and AMOVA analyses showed population structure among the ramp sites. STRUCTURE analyses were performed on both the entire dataset and the clone-corrected data set, and in both cases the log probability (ln[Pr(X/K)]) [48], was maximized at ΔK = 2, indicating two genetic groups (Fig 2A). One group, shown in orange, includes all MLGs sampled at *A. burdickii* sites while the second group, shown in blue, contains only MLGs from *A. tricoccum* sites, except for one MLG collected at *A. burdickii* site CC, which could have been a misidentified white color morph of *A. tricoccum* (Fig 2B). MLGs collected at *A. burdickii* sites showed little evidence of admixture, but evidence of admixture was present at some *A. tricoccum* sites, notably sites where *A. burdickii* co-occurs (9759, 9770, 9776, ECT1, ECT2, EF2) and in southern sites (PU, RPT, SFT, Figs 1B and 2B). Interestingly, some MLGs collected at *A. tricoccum* sites contained MLGs which clustered with the orange group, with little evidence of admixture. These included sites where *A. burdickii* is present (9770, 9776, ECT1, ECT2, EFT) and sites where it is not known to be present (TH, TRT, PU, TRH). Site ECUN, which appeared to be a mixed collection of *A. tricoccum* and *A. burdickii* based on voucher specimens, contained MLGs clustering in both blue and orange groups and admixed MLGs. While Bayesian STRUCTURE analysis, due to its assumption of Hardy-Weinberg equilibrium, may not be the most appropriate method for examining genetic structure in a partially clonal species [55], it did show that MLGs obtained from *A. burdickii* populations all clustered into one genetic group (orange) and these genotypes showed little admixture with the second genetic group (blue, Fig 1B and Fig 2B).

**Fig 3:**
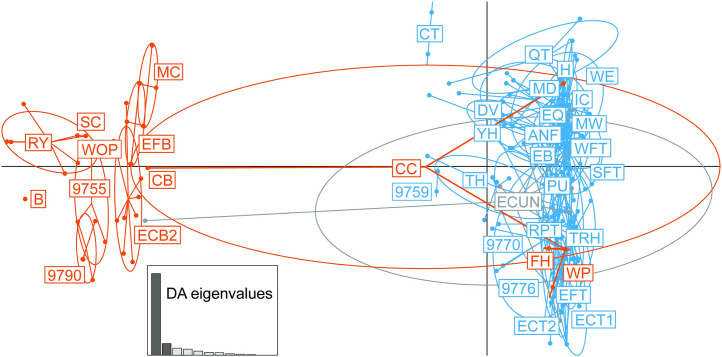
DAPC plot of clone-corrected data from collections which had ten or more genotyped ramps. MLGs form two clusters corresponding to *A. burdickii* (left cluster) and *A. tricoccum* (right cluster). Dots represent MLGs, sites identified as *A. burdickii* at collection are shown in orange, and sites identified as *A. tricoccum* at collection are shown in blue, ECUN which appeared to be a mixed collection from voucher specimens, is shown in grey.

Consistent with the STRUCTURE findings, DAPC analysis of the clone-corrected data from populations with n = 10 or more genotyped ramps showed two clusters. After 20 principal components were retained, one cluster contained only MLGs from *A. burdickii* sites while the second cluster contained all the MLGs from *A. tricoccum* sites (Fig 3). *A. burdickii* site CC contained three MLGs, one MLG which was shared by 82% of the CC individuals clustered with *A. burdickii*, and two MLGs representing 6% and 12% of CC individuals clustered with *A. tricoccum*. This further supports the STRUCTURE findings that some misidentified *A. tricoccum* individuals may have been collected at this site. Inconsistent with the STRUCTURE results, DAPC placed two *A. burdickii* sites (FH and WP) with the *A. tricoccum* cluster, whilst no *A. tricoccum* collections were found in the *A. burdickii* cluster. Site ECUN which appeared to be a mixed collection of *A. burdickii* and *A. tricoccum* based on voucher specimens, was placed in the *A. tricoccum* cluster by DAPC, but contained one MLG that was placed in the *A. burdickii* cluster. Sensitivity analysis showed the clustering observed in STRUCTURE and DAPC remained consistent even when the AT04 marker was dropped from the analyses (S5 Fig). Consistent with the previous analyses, AMOVA showed that approximately 27.29% of the overall genetic variation is found between *A. burdickii* and *A. tricoccum* site groups and high differentiation is present between the two groups (F_CT_ = 0.27293, p < 0.00001, Table 3). Substantial percentages of overall diversity and high differentiation was also partitioned among populations within groups (33.89%, F_SC_ = 0.46607, p < 0.00001) and within populations (38.82%, F_ST_ = 0.61180, p < 0.00001).

**Table 3:** AMOVA analysis of *A. burdickii* and *A. tricoccum* (non-clone-corrected data).

Source of variation	Degrees of freedom	Sum of squares	Variance components	Percentage of variation (%)	F-statistic
Between groups *A. burdickii* vs *A. tricoccum* *sites*	1	228.665	0.41698	27.29	F_CT_ = 0.27293*
Among populations within groups	35	557.366	0.51773	33.89	F_SC_ = 0.46607*
Within populations	1061	629.274	0.59310	38.82	F_ST_ = 0.61180*
Total	1097	1415.305	1.52780	100.00	

F_ST_ fixation index within populations; F_SC_ fixation index among populations within groups; F_CT_ fixation index between groups.

*p < 0.00001.

**Fig 4:**
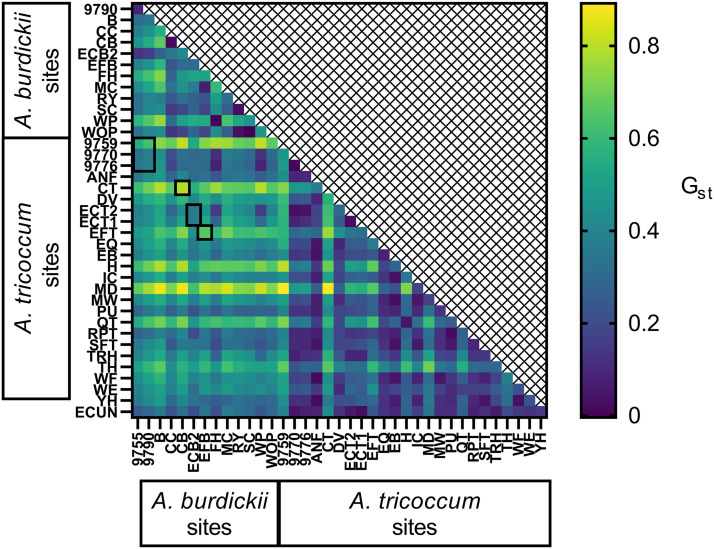
Pairwise G_st_ values for sites with ten or more genotyped individuals (non-clone- corrected data). Cooler colors indicate lower G_st_ and less differentiation. G_st_ values between *A. burdickii* and *A. tricoccum* collections at the same sites are highlighted by the black boxes.

Pairwise G_st_ values were calculated for all sites with ten or more individuals using the non-clone-corrected data (Fig 4). At the four sites where both *A. tricoccum* and *A. burdickii* cooccur, the average pairwise G_st_ between the *A. burdickii* and *A. tricoccum* collections was 0.560 and varied from 0.362 (ECB2 vs ECT1 and ECT2) to 0.790 (CB vs CT) indicating significant differentiation (p ≤ 0.001 for all comparisons and significant after Bonferroni correction) at these locations between the *A. burdickii* and *A. tricoccum* collections. Mean pairwise G_st_ for *A. burdickii* sites was 0.367 versus 0.275 for *A. tricoccum* sites. Raw genotypic data (individual-by-locus allele scores) are included in the supporting information (S2 File).

## Discussion

### Ramp genetic diversity and clonality

Despite the growing interest in ramp foraging [13] and the emerging conservation concerns regarding overharvesting of wild populations, little is known about ramp genetic diversity. This information could be valuable to identify germplasm conservation priorities and contribute to future selection and breeding work. To address this need, we developed a set of 18 microsatellite markers and used them to genotype *A. burdickii* and *A. tricoccum* accessions collected from midwestern and northeastern sites comprising a significant portion of the ramp’s range in the United States. Only four of the 18 markers were polymorphic (22.2%), which is notably lower than for other Alliums including *A. cepa* L. (95.2%, [56]), *A. fistulosum* (66%, [37]), and *A. sativum* (62.5%, [57]). Due to the limited number of polymorphic markers, we were able to isolate, we recommend that genotyping-by-sequencing methods (e.g., MIG-Seq, [58]) be developed and deployed for future studies of ramp genetic diversity.

We found that *A. burdickii* populations are more clonal and less diverse than *A. tricoccum*, but that ramp populations, especially among *A. tricoccum*, can vary considerably in clonality and diversity. Although these estimates could be biased since only four polymorphic markers were used. It has been established that bulb division is the primary mode of population increase in ramps [19]. One possible explanation of our results is that sexual reproduction and seedling recruitment, even if they contribute negligibly to population increase, may be reduced in *A. burdickii* populations compared to *A. tricoccum*. Six out of 13 (46%) *A. burdickii* sites had fewer than three MLGs while three out of 24 (12.5%) *A. tricoccum* sites had fewer than three MLGs. Furthermore, the mean eMLG for *A. tricoccum* (7.16) was more than double that of *A. burdickii* (2.96). Asexual reproduction is widespread in ramps; samples were collected in a manner to avoid collecting clones, but clones were detected in all *A. burdickii* populations and the majority of *A. tricoccum* populations including the most diverse site sampled (PU, Hexp = 0.567). However, only four microsatellite markers were used to discern the genotypes so it is possible that clonality may be overestimated and therefore the differences we observed between *A. burdickii* and *A. tricoccum* sites should be confirmed using genotyping-by-sequencing.

Notably, field observations by Jones [30] and field and common garden experiments by Sitepu [33], along with our own observations, agree that *A. burdickii* produces fewer flowers per umbel than *A. tricoccum*, reducing the potential for sexual reproduction on a per plant basis for *A. burdickii* compared to *A. tricoccum*. *A. tricoccum* also produces larger seeds than *A. burdickii* [30,33] which could lead to increased germination and seedling establishment in *A. tricoccum* versus *A. burdickii.* Furthermore, *A. burdickii* leaves emerge later than *A. tricoccum* and undergo senescence before leaves of *A. tricoccum* [27,28,30,33], giving *A. burdickii* a shorter window for photosynthesis, bulb growth, and nutrient storage compared to *A. tricoccum*. *A. burdickii* bulbs are smaller than *A. tricoccum* [30,33] and therefore may have less resources available for flowering and fruiting compared to *A. tricoccum*. Also, *A. burdickii* flowers two to three weeks earlier than *A. tricoccum* and for a shorter period [28,33,59], which may result in less opportunity for sexual reproduction in *A. burdickii*. Earlier fruiting in *A. burdickii* could also lead to increased seed predation compared to *A. tricoccum*, which could further limit seedling recruitment. Habitat differences between *A. burdickii* and *A. tricoccum* may also favor increased sexual reproduction in *A. tricoccum*. *Allium tricoccum* prefers moister conditions than *A. burdickii* (32, 34, 60] and soil moisture has been shown to be an important factor in ramp growth and reproduction [61–63]. While mature *A. burdickii* bulbs are more deeply set and rooted than *A. tricoccum*, which may allow it to survive in areas of lower soil moisture than *A. tricoccum*, seed germination and seedling establishment and survival could be lower in these areas than in the moister soils preferred by *A. tricoccum*. Additional demographic and reproductive studies of both *A. burdickii* and *A. tricoccum* are needed to confirm our hypothesis of differential sexual reproduction and seedling recruitment in these species.

We found that ramp populations vary considerably in clonality and genetic diversity. One large *A. tricoccum* population (MD) was entirely clonal while no clones were detected in three *A. tricoccum* populations (ANF, RPT, WE), all of which had moderate levels of genetic diversity (Hexp ranged from 0.439–0.560). Our sampling strategy and limited number of markers did not allow us to assess how clonality and diversity is spatially distributed within individual populations but these are important studies to do in the future, especially in relation to ramp density and harvest pressure. Further research is also needed to understand what site factors might be associated with increased diversity and/or less clonality at sites. Abiotic factors such as soil fertility, soil moisture, aspect, and light availability all contribute to photosynthetic capacity, bulb growth, and subsequent nutrient sequestration in the bulb, which can influence flowering, scape survival, and seed production [19,61–63]. Furthermore, significant yearly variation in seed production and seedling recruitment was observed in a dense population of *A. tricoccum* over a 5-year period at a site in Quebec, including a single mast year when reproductive output was greatly increased after favorable spring conditions [19]. Temporal and spatial differences in soil moisture, plant density, competition, and flower and seed predation could also impact the establishment of new genotypes. Disturbance in space and time can impact the clonal structure of populations by temporarily reducing competition [64–65]. Therefore, low to moderate levels of disturbance, such as careful harvesting and thinning of dense patches could create opportunities for seedling recruitment and increase diversity [64–66]. However more extensive disturbance (e.g., digging large patches, fire, logging, extended drought) could lead to reduced diversity if mortality is high and seedling recruitment is low [64–65]. It is worth noting that the extensive *A. tricoccum* site MD, which we found to consist of a single MLG has historically been harvested heavily each spring, while the sites with the highest genetic diversity (ANF, SFT, RPT, PU) are in protected areas which either prohibit or limit ramp harvesting.

### Ramp taxonomy

Despite long established differences in morphology, habitat, and phenology between *A. burdickii* and *A. tricoccum* [27,28,30,33,59,60], there is still debate over whether *A. burdickii* should be considered a separate species from *A. tricoccum* [20,26,31,32]. Our study provides the first preliminary genetic support for *A. burdickii* and *A. tricoccum* as two distinct species. Both Bayesian STRUCTURE analysis (which assumes HWE) and DAPC (which does not assume HWE) indicate two primary genetically differentiated sets of populations of ramps and that the assignment of MLGs into these genetic populations aligns closely with our species assignments at sample collection, which were based on established morphological and phenological differences between the two species [30,33]. STRUCTURE did place some *A. tricoccum* collections with the *A. burdickii* group and suggested potential admixture in populations; however, this was not the case with DAPC analysis where MLGs clearly clustered into two groups. While we are cautious to not overinterpret the STRUCTURE results, given it may not be the most appropriate tool for examining structure in clonal populations [55], it has been proposed based on morphological, habitat, and phenological differences, that additional green ramp taxa may exist [33], some which have more morphological overlap with *A. tricoccum* and could have been potentially collected in this study. DAPC did place two *A. burdickii* collections in the *A. tricoccum* cluster (though STRUCTURE did not). These two populations (WP, FH) are located near each other and were identified as *A. burdickii* based on morphological criteria by four of the authors. Additional markers or alternative genetic methods may help clarify some of these inconsistencies. High pairwise G_st_ values between *A. burdickii* and *A. tricoccum* collections found at the same sites, and AMOVA showing substantial genetic variation and high differentiation between the taxa further support *A. burdickii* as a distinct species. It should be noted that sampling of southern ramp populations was limited in this study and therefore may have affected interpretations of geographic genetic structure and admixture. While our conclusions rest on a small number of microsatellite loci and primarily on populations from the northern half of the range of the complex, the signal of genetic differentiation is quite strong. Undoubtedly more genetic markers, denser sampling of geographically separate populations across all taxa, and comprehensive geographic sampling across the ranges of all taxa (especially the southern populations), would provide greater insights into differences in genetic diversity and distribution of genetic variation within and among populations of the taxa. However, it is not likely to reverse the clear pattern of genetic differentiation into two ramps taxa shown here, and sampling in the southeastern and central Appalachian regions may provide further genetic evidence to support additional taxa detected by Sitepu. A more locus-rich genetic approach, such as Mig-SEQ (58), might yield new information regarding the evolutionary status of the intermediates and finer-scale population genetic structure and should also be done to confirm the preliminary genetic results presented here, given the low number of genetic markers used in the study and that the possibility of null alleles could not be ruled out.

Differences in phenology may contribute to reproductive isolation, as *A. burdickii* flowers with high consistency one or more weeks earlier than *A. tricoccum,* a trait first observed by Jones [30] and confirmed by Sitepu [33] and by citizen science data collected through iNaturalist [59]. Some overlap in flowering phenology may occur in late June and early July at certain sites [30] but it is unknown whether the species are interfertile. Additionally, while they sometimes co-occur, *A. burdickii* tends to occur on drier, more upland sites compared with *A. tricoccum* [30,60].

### Guidance for conservation

We recommend that *A. burdickii* be recognized as a distinct species and should be tracked separately from *A. tricoccum* by land managers and botanical organizations. Widespread harvesting of *A. burdickii* has not been reported but the overall low diversity of populations and high clonality suggest populations could benefit from human-assisted seed sowing to promote the establishment of new genotypes. *Allium tricoccum* populations varied greatly in terms of clonality and genetic diversity, though even the most diverse *A. tricoccum* populations are only moderately diverse. Therefore, ramp harvesting strategies that minimize the loss of genetic diversity or promote diversity should be encouraged. Such strategies include limiting harvest to bulbs that are undergoing bulb division (e.g., take one, leave one); careful harvesting from dense patches to create patch gaps to promote seedling recruitment (e.g., thinning); transplanting ramps from dense patches to other areas; and collecting and sowing seeds in patch gaps or lower density areas to encourage seedling recruitment. Furthermore, sites we identified that are enriched with unique MLGs might be considered/prioritized for seed banking efforts.

## Conclusion

Our data using microsatellite markers, applied primarily to northeastern and midwestern populations, show for the first time that there are two genetic lineages of ramps which correspond to *A. burdickii* and *A. tricoccum*. Our data do not support the existence of additional ramp taxa but the limited number of polymorphic markers we found may have underestimated population structure. Reproductive isolation could be maintained in *A. burdickii* due to differences in phenology, habitat, and reduced sexual reproduction compared to *A. tricoccum.* We found that ramp populations vary considerably in clonality and genetic diversity, though our estimates may be impacted by the low number of markers used to genotype and that we cannot rule out the possibility of null alleles. Ramp harvesting and stewardship practices that minimize the loss of diversity or increase diversity, such as thinning to promote sexual recruitment and seed sowing, should be promoted. Finally, while microsatellites have been successfully employed in many plant species to measure genetic diversity, due to the low number of polymorphic markers we found, other methods such as genotyping-by-sequencing should be developed and used in future genetic studies of ramps to confirm and expand on the results presented in this manuscript

## Supporting information

S1 Fig Key morphological differences between *A. tricoccum and A. burdickii.* Based on Sitepu [37].
(XLSX)

S2 Fig Histogram of Pairwise Genetic Distances. (DOCX)

S3 Fig Multi-locus genotype summary by collection (site). (PDF)

S4 Fig Unique multi-locus genotypes summary. A. Number of unique multi-locus genotypes (MLGs) and B. number of unique MLGs by site for *A. tricoccum* (B) and *A. burdickii* (C) collections.
(TIF)

S5 Fig Sensitivity/locus drop analysis on clone-corrected MLGs. (JPG)

S1 Table Microsatellite Loci and Primer Information. (DOCX)

S2 Table Collection and Voucher Information. (DOCX)

S3 Table Linkage disequilibrium summary for each collection for both clone-corrected and non-clone-corrected data. (XLSX)

S1 File Hardy-Weinberg Equilibrium and null alleles tests. (XLSX)

S2 File Raw genotypic data and associated metadata. (XLSX)
