# Supplementary material for: New microsatellite markers distinguish two species of ramps (Allium tricoccum Aiton Complex, Amaryllidaceae) and show variation in clonality and genetic diversity between species and among populations
Source: PLoS One. 2025 Oct 8;20(10):e0332086. doi: 10.1371/journal.pone.0332086 (PMC12507257; doi:10.1371/journal.pone.0332086)
Supplement: S2 Fig — (DOCX) [file pone.0332086.s002.docx]

Fig. S2 Histogram of Pairwise Genetic Distances used to identify the cutoff for defining clones. Note the inflection in the bar graph between 0.05 and 0.01 (red arrow).
