# Supplementary figures and images for: New microsatellite markers distinguish two species of ramps (Allium tricoccum Aiton Complex, Amaryllidaceae) and show variation in clonality and genetic diversity between species and among populations
Source: PLoS One. 2025 Oct 8;20(10):e0332086. doi: 10.1371/journal.pone.0332086 (PMC12507257; doi:10.1371/journal.pone.0332086)

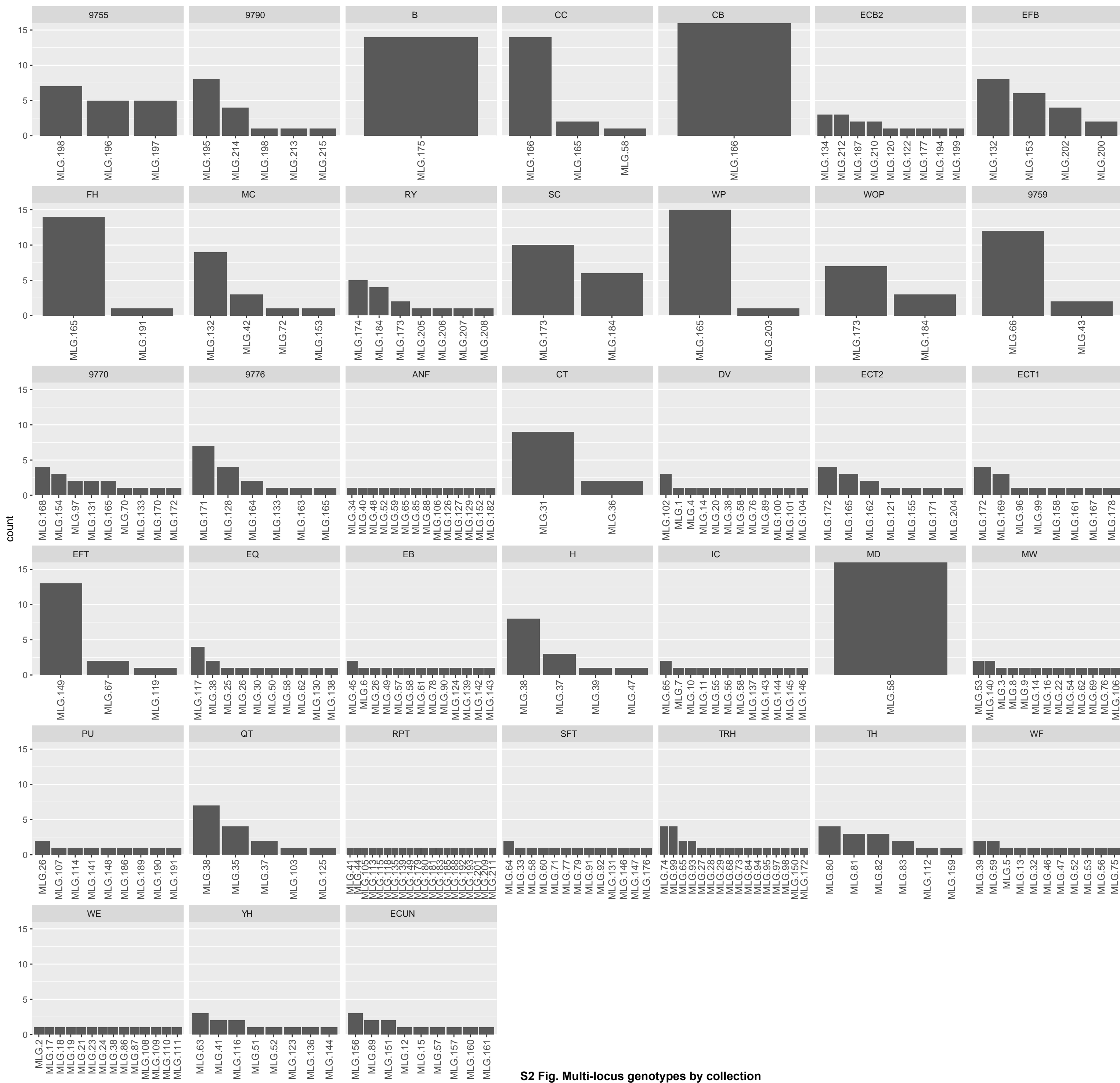

S2 Fig. Multi-locus genotypes by collection

Supplement: S3 Fig — (PDF) [file pone.0332086.s003.pdf]

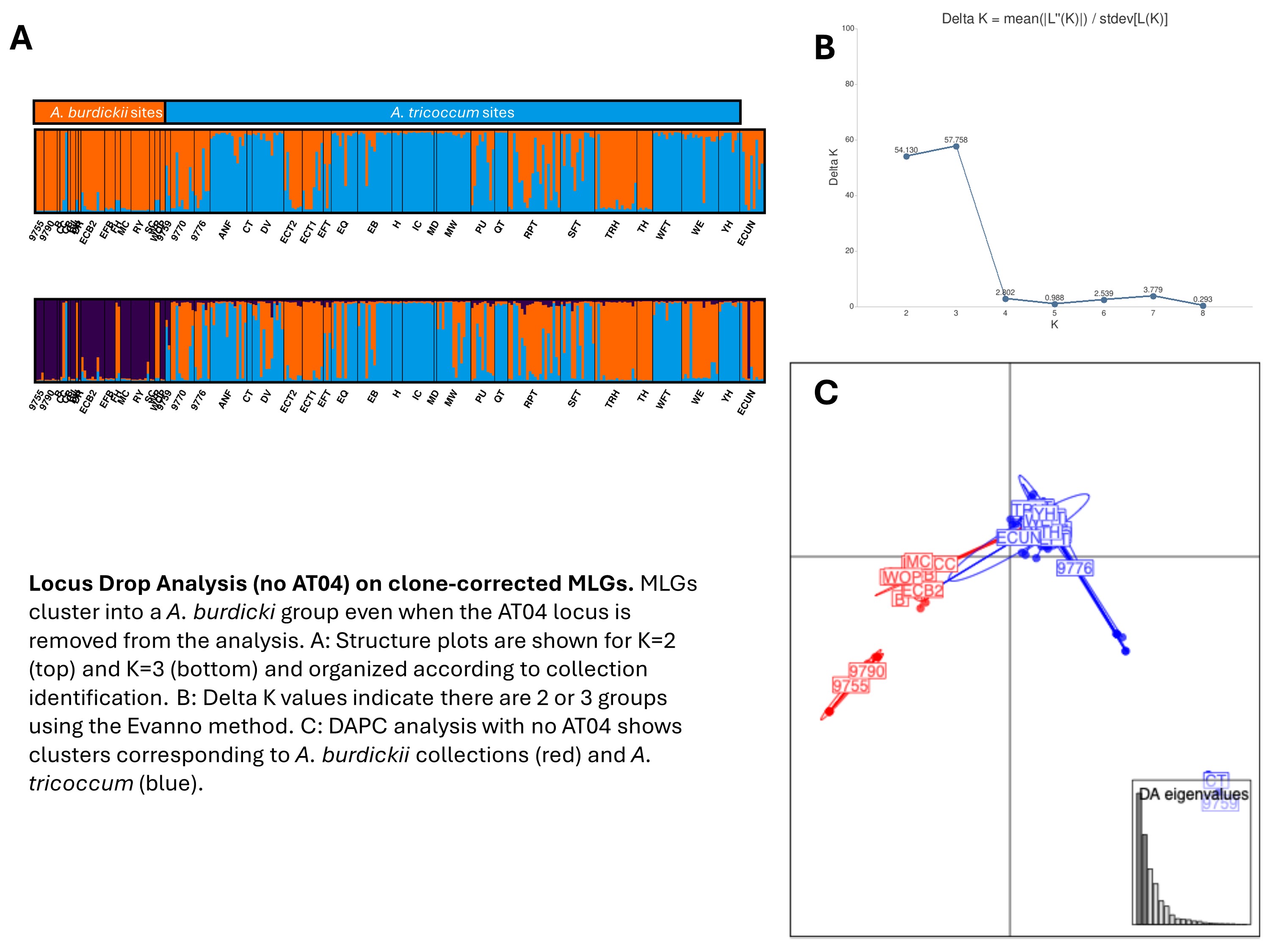

Supplement: S5 Fig — (JPG) [file pone.0332086.s005.jpg]
