# Supplementary material for: New microsatellite markers distinguish two species of ramps (Allium tricoccum Aiton Complex, Amaryllidaceae) and show variation in clonality and genetic diversity between species and among populations
Source: PLoS One. 2025 Oct 8;20(10):e0332086. doi: 10.1371/journal.pone.0332086 (PMC12507257; doi:10.1371/journal.pone.0332086)
Supplement: S1 Table — (DOCX) [file pone.0332086.s006.docx]

**S1 Table Microsatellite Loci and Primer Information**

| **Locus** | | **F Primer sequence (5' to 3')** | **R Primer sequence (5' to 3')** | **Motif** | **Size range** |
| --- | --- | --- | --- | --- | --- |
| AT01 | CGGACCTCGTATGCACAAG | | GTTTAGGGTACTGTTCATAGGCGG | AAAT | 110 |
| AT02 | GGAGTGTAATTTGCGGCATTG | | GTTTGGGTGAAATGGAGAAGGC | AAC | 116 |
| **AT04** | **ATCTGGTTCGGGCATTCAAC** | | **GTTTGGTTCGACAGTGGTTGG** | **AAG** | **111-117** |
| AT07 | GGAGTGAGAAACGTGATGGG | | GTTTGGGTGGGTTCATTTATTTGGC | AATT | 127 |
| AT10 | AGTGAGTACGATCAGGCATTG | | GTTTCCAAATCGATCCGTTCCC | AC | 129 |
| **AT32** | **GCAAACAAATCATGGCCATCC** | | **GTTTGGTGGAACGATGTGGAGTATC** | **AGG** | **93-105** |
| AT37 | GGGATTGTTGAGAAAGAAACCG | | GTTTGCAAAGCCGAACTAGGTC | AT | 220 |
| AT50 | ACGAGCTAAGTGTCGATCGC | | GTTTCGACTTTCACTTGGATCGG | AAT | 255 |
| AT51 | AGACGCTAGCTTGAGACTGG | | GTTTAAGCGAACTCAGAAATTCCAAC | AAAT | 168 |
| **AT55** | **AGCAACATCTCCTTTGCGTG** | | **GTTTCGAGCGAGCAATGAGAAAGG** | **AC** | **221-237** |
| AT57 | AGTCCTCATACCTGCACCTG | | GTTTACTTTGGTGTGTCATGTCGG | AC | 190 |
| **AT58** | **GTTTACTCTTCTCCTGCACGCAC** | | **CCCGAAACCTTTGCACAGTAG** | **AC** | **100-116** |
| AT59 | TGGTTCCCGTTCCAATTTCC | | GTTTGGACGTATTGAGAGAGCGTAC | AC | 198 |
| AT66 | CCCGGGTCAGACTTGTGAG | | GTTTGTGTATGGGAGGAGAGCG | AC | 122 |
| AT88 | GTTTGGGCTACTCTTCTAATCGGC | | AGAACCGAGGCTCAACAGAG | AT | 150 |
| AT89 | TTGGTCAACTTTCGCCCAAG | | GTTTGCTGCAGGAGAAGAGC | AT | 137 |
| AT95 | GTTTCATCAAATTCAATGCCGAGTG | | GGTGATTGACAATACGCTACAC | AT | 145 |
| AT96 | GCAGTGAACAGACAGGTGAG | | GTTTCCAATCGATGTGGCTTCAAG | AT | 224 |
| **Bold font indicates polymorphic loci and the primers used for genotyping** | | | | | |
