# Supplementary material for: New microsatellite markers distinguish two species of ramps (Allium tricoccum Aiton Complex, Amaryllidaceae) and show variation in clonality and genetic diversity between species and among populations
Source: PLoS One. 2025 Oct 8;20(10):e0332086. doi: 10.1371/journal.pone.0332086 (PMC12507257; doi:10.1371/journal.pone.0332086)
Supplement: S2 Table — (DOCX) [file pone.0332086.s007.docx]

| Collection | US State | Species | Voucher location | Voucher ID | Samples Collected and  Identified by |
| --- | --- | --- | --- | --- | --- |
| ANF | PA | *A. tricoccum* | CM | In progress | Authors |
| B | OH | *A. burdickii* | CM | CM543935 | Authors |
| CB^1^ | MN | *A. burdickii* | CM | CM564878 | Land manager |
| CT^1^ | MN | *A. tricoccum* | CM | CM564876 | Land manager |
| CC | PA | *A. burdickii* | CM | In progress | Authors |
| CJ | PA | *A. burdickii* | CM | CM543419 | Authors |
| DH | PA | *A. burdickii* | CM | CM543483 | Authors |
| DR | WV | *A. burdickii* | CM | CM543926 | Authors |
| DV | PA | *A. tricoccum* | CM | In progress | Authors |
| EB | PA | *A. tricoccum* | CM | In progress | Authors |
| ECB2^2^ | IN | *A. burdickii* | CM | CM564881 | Land manager |
| ECT1^2^ | IN | *A. tricoccum* | CM | CM564880 | Land manager |
| ECT2^2^ | IN | *A. tricoccum* | CM | CM564879 | Land manager |
| ECUN^2^ | IN | unknown/mixed | CM | CM564882 | Land manager |
| EFB^3^ | PA | *A. burdickii* | CM | CM543477 | Authors |
| EFT^3^ | PA | *A. tricoccum* | CM | CM543479 | Authors |
| EQ | PA | *A. tricoccum* | CM | In progress | Authors |
| FH | PA | *A. burdickii* | CM | CM543932 | Authors |
| H | PA | *A. tricoccum* | CM | CM543416 | Authors |
| IC | PA | *A. tricoccum* | CM | In progress | Authors |
| MC | PA | *A. burdickii* | CM | CM543947 | Authors |
| MD | PA | *A. tricoccum* | CM | CM543417 | Authors |
| 9755^4^ | MN | *A. burdickii* | CM | CM564886 | Land manager |
| 9759^4^ | MN | *A. tricoccum* | CM | CM654887 | Land manager |
| 9770^4^ | MN | *A. tricoccum* | CM | CM564889 | Land manager |
| 9776^4^ | MN | *A. tricoccum* | CM | CM564888 | Land manager |
| 9790^4^ | MN | *A. burdickii* | CM | CM564885 | Land manager |
| MW | PA | *A. tricoccum* | CM | CM543423 | Authors |
| PU | NC | *A. tricoccum* | No voucher due to site restrictions | | |
| QT | PA | *A. tricoccum* | CM | CM543420 | Authors |
| RPT | TN | *A. tricoccum* | No voucher due to site restrictions | | |
| RY | PA | *A. burdickii* | CM | CM543938 | Authors |
| SC | PA | *A. burdickii* | CM | CM543475 | Authors |
| SFT | WV | *A. tricoccum* | BHO | BHO060002 | Authors |
| TH | TN | *A. tricoccum* | BHO | BHO060003, BHO060004 | Authors |
| TRH | NC | *A. tricoccum* | No voucher due to site restrictions | | |
| WE | PA | *A. tricoccum* | CM | In progress | Authors |
| WFT | WV | *A. tricoccum* | BHO | BHO060005 | Authors |
| WOP | PA | *A. burdickii* | CM | CM543929 | Authors |
| WP | PA | *A. burdickii* | CM | In progress | Authors |
| YH | WV | *A. tricoccum* | BHO | BHO060006, BHO060007 | Authors |

Collections with the same subscript were collected at the same location. Carnegie Museum of Natural History Herbarium (CM) Pittsburgh, PA USA, Bartley Herbarium of Ohio University (BHO) Athens, OH USA, Penn State University Herbarium (PAC) University Park, PA USA.
